# Intrafamilial Transmission of Methicillin-Resistant *Staphylococcus aureus*^1^

**DOI:** 10.3201/eid1510.081532

**Published:** 2009-10

**Authors:** Sabrina A. Pozzi Langhi, James O. Robinson, Julie C. Pearson, Keryn J. Christiansen, Geoffrey W. Coombs, Ronan J. Murray

**Affiliations:** Royal Perth Hospital, Perth, Western Australia, Australia (S.A. Pozzi Langhi, J.O. Robinson, J.C. Pearson, K.J. Christiansen, G.W. Coombs, R.J. Murray; Curtin University of Technology, Perth (K.J. Christiansen, G.W. Coombs).

**Keywords:** MRSA, intrafamilial transmission, decolonization, community-acquired, necrotizing pneumonia, antimicrobial resistance, staphylococci, letter

**To the Editor:** Community-acquired methicillin-resistant *Staphylococcus aureus* (CA-MRSA) infection was first described in our region over 15 years ago (*1*). More recently, CA-MRSA has become a global concern and is now a common cause of skin and soft tissue infections in the United States (*2*). An association between severe CA-MRSA infection (e.g., necrotizing fasciitis and pneumonia) and the synergohymenotrophic exotoxin Panton-Valentine leukocidin (PVL) has been made (*3*,*4*). Reports have documented CA-MRSA transmission among household members; however, most cases have been mild or moderate infections or asymptomatic colonization (*5*–*7*). We describe intrafamilial transmission of a PVL-containing CA-MRSA clone common in Australia (ST30-MRSA-IV) between a nurse who suffered recurrent abscesses and her husband, who died of severe pneumonia.

In July 2006, a 61-year-old previously healthy nurse (Mrs A) sought treatment for an infected seborrheic cyst of the scalp. Culture of pus yielded MRSA that was susceptible to clindamycin. She was treated with oral clindamycin. After resolution of the infection, topical MRSA decolonization therapy with 3% hexachlorophane body wash (daily), 20% cetrimide shampoo (3×/wk), and 2% mupirocin nasal ointment (3×/d) was administered for 10 days, as per our institutional protocol for MRSA-colonized healthcare workers. Subsequently, MRSA surveillance swabs from the nose, throat, and scalp obtained weekly for 10 weeks and cultured on selective MRSA chromogenic agar and in selective broth enrichment media were negative. Household members were not screened for MRSA colonization.

Six months later, in January 2007, the patient’s husband (Mr A), a 60-year-old smoker who was her only household contact, was admitted with a 1-day history of dyspnea, pleuritic chest pain, cough with sputum, fever, vomiting, and diarrhea. On admission, he was unwell, with tachycardia (pulse rate 132 bpm), hypotension (95/60 mm Hg), tachypnea (40 breaths/min), and hypoxia (oxygen saturation 93% on 15 L O_2_/min). A chest radiograph showed bilateral infiltrates and a right pleural effusion. He was diagnosed with community-acquired pneumonia and treated with intravenous ceftriaxone and azithromycin as per local protocol. However, within 12 hours, his condition deteriorated, necessitating admission to the intensive care unit for ventilation and inotropic support. Broncho-alveolar lavage (BAL) fluid demonstrated gram-positive cocci in tetrads, and intravenous vancomycin and dicloxacillin were added to therapy. Despite aggressive supportive measures, Mr A’s condition continued to deteriorate, and he died 28 hours after admission. MRSA was subsequently cultured from blood, sputum, and BAL fluid; an autopsy was not performed.

In June 2007, Mrs A sought treatment for an abscess with cellulitis on the left thigh. The abscess was surgically drained, and cultures again yielded MRSA. She was treated with intravenous and oral clindamycin for 10 days and subsequently underwent repeat MRSA decolonization therapy; again, swabs taken 1×/wk for 10 weeks postdecolonization were negative.

Molecular typing of the MRSA isolates obtained from Mrs A at the time of her initial skin infection, Mr A’s blood culture, and Mrs A’s second skin infection was performed by using contour-clamped homogenous electric field electrophoresis (CHEF) according to a previously described method (*8*) (Figure). The CHEF patterns were indistinguishable and were identical to the known CHEF pattern for ST30-MRSA-IV (*9*). All 3 isolates contained the *lukF-PV/lukS-PV* genes that encode PVL and had the same antibiogram (i.e., isolates were resistant only to β-lactam antimicrobial agents).

**Figure:**
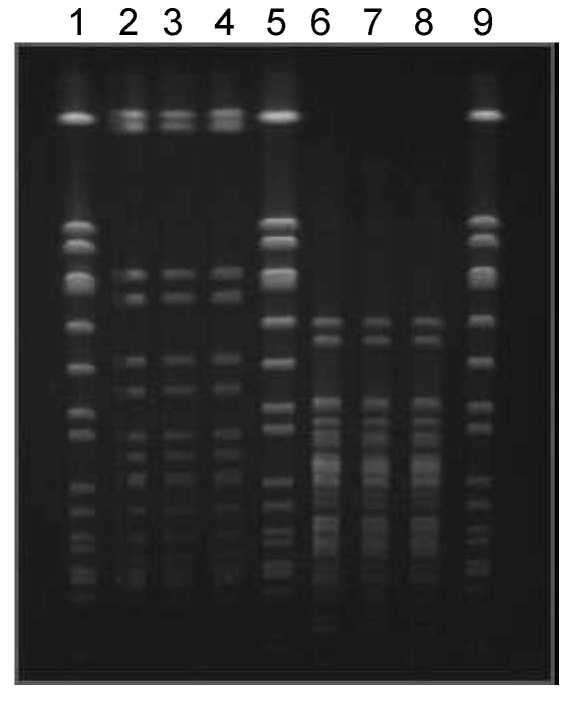
Contour-clamped homogenous electric field electrophoresis of *Staphylococcus aureus* isolates. Lanes 2, 3, and 4 *(Sma*1 restriction): methicillin-resistant *S. aureus* (MRSA) isolated from Mrs A’s first infection, Mr A’s blood culture, and Mrs A’s second infection, respectively. Lanes 6, 7, and 8 (*Apa*1 restriction): MRSA isolated from Mrs A’s first infection, Mr A’s blood culture, and Mrs A’s second infection, respectively. Lanes 1, 5, and 9: *S. aureus* NCTC8325.

We describe intrafamilial MRSA transmission (defined as >2 family members who live at the same postal address and who are colonized or infected with a MRSA strain having the same CHEF pattern) that resulted in a fatal outcome. The MRSA strain responsible (ST30-MRSA-IV, or Western Samoan phage pattern/Oceania strain MRSA) is a common cause of CA-MRSA infection in Australia.

Recurrent MRSA infection developed in Mrs A several months after completion of apparently successful MRSA decolonization therapy. We could not determine whether this recurrence was because of persistent MRSA colonization not detected by surveillance (e.g., perineal or gastrointestinal colonization) or whether Mrs A was successfully decolonized but Mr A’s colonization/infection resulted in recolonization and subsequent infection. Whatever the explanation, this case highlights a potential weakness in MRSA surveillance programs that rely on short-term, limited-site surveillance.

A comprehensive MRSA search-and-destroy policy in place for over 25 years has prevented MRSA from becoming endemic in our institution (*10*). However, the rapidly changing epidemiology of MRSA in becoming a predominantly community pathogen represents a significant challenge to the ongoing success of this policy. In response to this challenge, the Western Australian Department of Health has implemented a community-based MRSA search-and-destroy program for patients with MRSA infection caused by exotic PVL-positive clones (e.g., ST30-MRSA-IV, ST93-MRSA-IV, ST80-MRSA-IV, and ST8-MRSA-IV/USA300). This program includes treatment/decolonization therapy for the index case, screening of household members for MRSA infection/colonization, and simultaneous treatment/decolonization if MRSA is identified. Although a similar approach has proved successful in Denmark (*6*), whether this success can be sustainable on a larger scale remains to be seen.
